# Effects of three Commercial Mouth Rinses on the Cultured Fibroblasts: An in Vitro Study

**Published:** 2013-06

**Authors:** J Ghabanchi, A Moattari, R Darafshi, A Andisheh Tadbir, H Khorshidi, M Shakib

**Affiliations:** a Dept. of Oral Medicine, School of Dentistry, Shiraz University of Medical Sciences, Shiraz, Iran.; b Dept. of Microbiology, School of Medicine, Shiraz University of Medical Sciences, Shiraz, Iran.; c Dept. of Prosthodontics, School of Dentistry, Shiraz University of Medical Sciences, Shiraz, Iran.; d Dept. of Oral and Maxillofacial Pathology, School of Dentistry, Shiraz University of Medical Sciences, Shiraz, Iran.; e Dept. of Periodontics, School of Dentistry, Shiraz University of Medical Sciences, Shiraz, Iran.; f Undergraduate Student, School of Dentistry, Shiraz University of Medical Sciences, Shiraz, Iran.

**Keywords:** Irsha, Chlorhexidine, Persica, Cytotoxicity, Mouthwash

## Abstract

**Statement of Problems: **An ideal antimicrobial agent should have minimal cytotoxic effect to host cells.

**Purpose:** The aim of this study was to determine the cytotoxic effect of three commercial mouthwashes (Chlorhexidine, Persica and Irsha) on the cultured fibroblasts.

**Material and Methods: **For determining the cytotoxic effect of Irsha, Chlorhexidine and Persica, uninfected cells were grown in the absence and presence of various concentration (2,4,8,16,32,64,128) of these mouth washes for 1, 2, 3 and 4 days.

**Results: **In this study, three mouth washes show cytotoxic effect on the cultured cells, at commercially available concentration and even diluted and Irsha was found to be the most toxic one. Cytotoxicity of three mouthwashes was reduced with decreasing concentration.

## Introduction

Good oral health has a major influence on an individual’s quality of life and health. Different systemic and chronic diseases can cause poor oral health and increase the incidence of oral diseases. The global demand for development of the new preventive and treatment methods and products which are safe, effective and economical is increased.

The maintenance of the oral health can be achieved, mainly, by mechanical and chemical means [1]. Among chemical agents, mouthwashes are widely used for personal oral hygiene because of their ability to inhibit dental plaque [2]. Plaque begins with the accumulation of Gram-positive streptococci, then increases with the deposition of gram-negative anaerobic bacteria [3].

The mouthwashes contain active agents in their chemical structure that may inhibit the microbial growth and the enzymatic reactions or may react directly with the volatile sulfur compounds to reduce their levels in the mouth [4]. Although mouthwashes have demonstrated the ability to inhibit the formation of biofilms, a little information is available on their genetic and cellular toxicity [5].

There are different chemical agents available commercially in the form of mouth rinses [6]. Among them, the most frequently used mouth rinses in Iran are: Chlorhexidine (CHX), Persica (extracted from *Salvadora Persica *Plant) and Irsha.

The aim of this study was to determine the cytotoxic effect of three mouthwashes on the cultured fibroblasts.

## Material and Methods

**Cell culture**

Vero cells (Fibroblast cells) were grown up in the 24-well plates (Nunc; Denmark) having Dulbecco’s modified eagle’s growth medium (DMEM; Sigma, USA) which contains 7% fetal bovine serum (Gidbco, Australian), 0.14% (v/v) sodium bicarbonate, 100 u/ml penicillin, 100g/ml streptomycin sulfate and 0.25g/ml amphotericin B. Then the plates were incubated at 37C under carbon dioxide (CO2) for 48 hours.

**Cytotoxicity assay**

Grown-up Vero-cell monolayers were washed twice with PBS. To determine the cytotoxicity of Persica, Irsha and Chlorhexidine; cells were grown in the absence and the presence of various concentrations (2, 4, 8, 16, 32, 64, 128) of these mouthwashes for 1, 2, 3 and 4 days. We observed the cells by microscope every 24 hours. The extent of cytotoxicity was confirmed by Trypan blue dye exclusion method. The 50 % cytotoxic concentration (CC50) was estimated by Karber method [7] and Chi-squared test was used for statistical analysis.

## Results

In this study, three mouthwashes demonstrated cytotoxic effect on the cultured cells at commercially available concentration and even diluted concentrations (up to 1:32). At the diluting concentration of 1:8; Irsha had more cytotoxicity than the other two mouthwashes (*p*= 0.02) and at 1:32 diluting concentration; Persica was less toxic than the CHX significantly (*p*= 0.01). 

**Figure 1:**
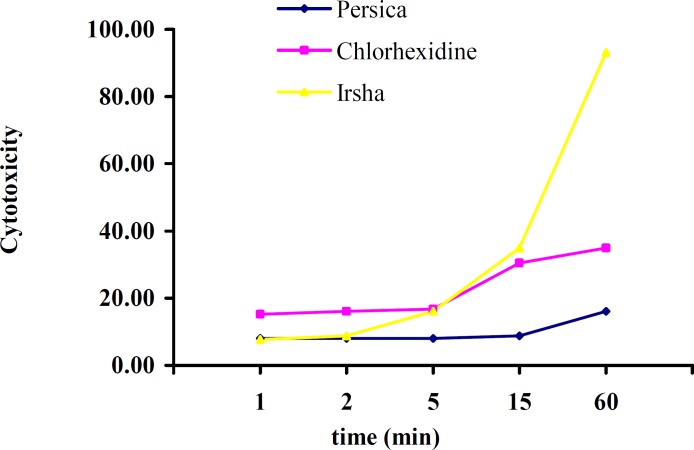
Comparison of the cytoxicity of three mouthwashes in different concentration at different times

Cytotoxicity of three mouth washes was reduced by decreasing the concentration (Figure 1).

## Discussion

In the present study; Irsha shows the most cytotoxic effect on the fibroblast cells. In the review of literature; we found only one study which investigated the antimicrobial effect of Irsha mouthwash [8]. In our previous study, we demonstrated its cytotoxicity on Hela cells [9].

Irsha mouthwash contains different components such as alcohol, glycerin, sodium lauryl sulfate (SLS), benzoic acid and allantoin.

Babich et al. [10] showed that SLS induced vacuolization in gingival fibroblasts. Cytotoxic activity of this mouth wash might probably have been due to the presence of SLS.

In the present study, it was found that Persica, at commercially available concentration, is cytotoxic to the fibroblast cells.

There were limited studies which investigated the cytotoxic effect of *Salvadora Persica* *(S.Persica).* In 1983, Mohammad and Turner evaluated the cytotoxic effect of the *S. Persica* plant on the oral tissues. They showed that fresh *S.Persica* Miswak had no cytotoxic effect but its usage after 24 hours had harmful components [11].

In the study conducted by Dormani et al.; minor side effects were seen on the male and female mice reproductive system after direct administration of high doses of *S. Persica* Miswak extract [12].

 Similar to our results, Rajabalian et al. [13] demonstrate that one hour exposure to a 0.1 % Persica solution caused irreversible cytotoxic effect on the cells that involved the process of wound healing but diluting solution with Fetal serum cuff (FCS) had protective effect against the drug cytotoxicity. They proposed that decreased cytotoxic effect of this mouthwash, when associated with FCS, is probably due to the interaction of its toxic components with serum proteins. Therefore, it seems that the toxic effects of Persica solution are because of the irreversible binding to the cellular proteins and impairment of their function [13]. 

In our study, similar to the Rajabalian et al. [13] study, cytotoxic effect of Persica mouthwash was reduced with the successive dilution, with the least toxic effect at 1:16, which may be due to interaction between FBS and Persica.

*S. Persica* contains different components such as Indole, Alkaloids, Flavonoids, the Sulphur-containing compounds, Tropaeolin and Phytosterol [14-15]. Cytotoxic activity of this mouthwash is probably due to the alkaloid and flavonoids components [13].

CHX is broadly used in dental practice to reduce plaque formation and gingivitis and also in controlling the root canal disinfection.However, information about its toxic effect, particularly in compare with other commercial mouthwashes, are contradictory [16].

In the current study, the cytotoxic effect of the CHX was more than Persica but less than Irsha. It was also found that CHX was cytotoxic to fibroblast cells depending on the concentration and contact time. This result was in accordance with many studies [17-18], which stated that CHX decreased the gingival fibroblasts proliferation in a dose dependent manner. 

In the different studies, CHX was stated to be toxic, even in low concentration, for variety of cell types such as epithelial cells, gingival fibroblasts, neutrophils, macrophages, and red blood cells in culture [17-19]. Moreover, in an animal study, it was stated that even topical application of CHX can result in its penetration through the epithelial barrier and therefore, triggering the tissue damage [20]. 

Chang et al. [21] examined the effects of CHX on cultured human periodontal ligament cells (PDL) cells in vitro and reported that CHX inhibited the protein synthesis in the human PDL cells. Faria et al. [22] showed that CHX caused two forms of cell death simultaneously in the fibroblast, the prevalence of apoptosis or necrosis depends on the intensity of the inciting stimulus (the concentration of CHX). 

The intrinsic mechanism underlying CHX-induced cytotoxicity in eukaryotic cells is, however, still unknown. It has been proposed that CHX inhibits the mitochondrial activity; protein and DNA synthesis and cell proliferation; causing cell death by ATP depletion [21, 23].

Our results showed all three solutions were toxic to the cultured fibroblasts with Irsha being the most cytotoxic mouthwash.

We suggest future studies to investigate the *in vivo *cytotoxicity of these three mouth washes.
